# Derivative Matrix-Isopotential Synchronous Spectrofluorimetry and Hantzsch Reaction: A Direct Route to Simultaneous Determination of Urinary δ-Aminolevulinic Acid and Porphobilinogen

**DOI:** 10.3389/fchem.2022.920468

**Published:** 2022-05-31

**Authors:** Muhammad Ajmal, Jia-Wen Wei, Yan Zhao, Yi-Hong Liu, Ping-Ping Wu, Yao-Qun Li

**Affiliations:** Department of Chemistry and the MOE Key Laboratory of Spectrochemical Analysis and Instrumentation, College of Chemistry and Chemical Engineering, Xiamen University, Xiamen, China

**Keywords:** matrix-isopotential synchronous fluorescence spectrometry, δ-aminolevulinic acid, porphobilinogen, simultaneous detection, derivative technique

## Abstract

Early and sensitive detection of δ-aminolevulinic acid (δ-ALA) and porphobilinogen (PBG) is the cornerstone of diagnosis and effective treatment for acute porphyria. However, at present, the quantifying strategies demand multiple solvent extraction steps or chromatographic approaches to separate δ-ALA and PBG prior to quantification. These methods are both time-consuming and laborious. Otherwise, in conventional spectrofluorimetry, the overlapping spectra of the two analytes cause false diagnosis. To overcome this challenge, we present a two-step approach based on derivative matrix-isopotential synchronous fluorescence spectrometry (DMISFS) and the Hantzsch reaction, realizing the simple and simultaneous detection of δ-ALA and PBG in urine samples. The first step is chemical derivatization of the analytes by Hantzsch reaction. The second step is the determination of the target analytes by combining MISFS and the first derivative technique. The proposed approach accomplishes following advantages: 1) The MISFS technique improves the spectral resolution and resolves severe spectral overlap of the analytes, alleviating tedious and complicated pre-separation processes; 2) First derivative technique removes the background interference of δ-ALA on PBG and vice versa, ensuring high sensitivity; 3) Both the analytes can be determined simultaneously via single scanning, enabling rapid detection. The obtained detection limits for δ-ALA and PBG were 0.04 μmol L^−1^ and 0.3 μmol L^−1^, respectively. Within-run precisions (intra and inter-day CVs) for both the analytes were <5%. Further, this study would serve to enhance the availability of early and reliable quantitative diagnosis for acute porphyria in both scientific and clinical laboratories.

## Introduction

Porphyrins play a pivotal role in chlorophyll, hemoglobin, and certain enzymes (Killiny et al., 2020). For human bodies, their synthetic sequence leads to the production of haem. However, any abnormality in the haem biosynthetic pathway can give rise to a class of genetic disorders, known as the porphyrias (Ajmal et al., 2019; Fujiwara et al., 2020; Jaramillo-Calle et al., 2021). The porphyrias are broadly categorized as either acute or non-acute. Amongst acute intermittent porphyria (AIP), hereditary coproporphyria (HP) and variegate porphyria (VP), AIP is the most common type of acute porphyria. It is caused by reduced activity of porphobilinogen deaminase (PBGD), which is the third enzyme in the haem pathway (Bechara et al., 2021). The disease (AIP) is characterized by deadly episodes of neurovisceral attacks featured by hypertension, autonomic dysfunction, and abdominal pain (Lin et al., 2011). As δ-aminolevulinic acid (δ-ALA) and porphobilinogen (PBG) are the well documented precursors for the biosynthetic pathway of haem, disorders of their urinary concentrations bear remarkable diagnostic and therapeutic significance for acute porphyria. However, unlike AIP, HP, and VP, where the urinary concentrations for both δ-ALA and PBG are raised, elevated concentration of δ-ALA alone without raising PBG marks the presence of diseases like ALA-D deficiency porphyria (ADP), hereditary tyrosinaemia type I, and lead toxicity (van’t Klooster et al., 2017). Although PBG concentration in urine is recommended as the first identification test for an acute attack (Herrick and McColl, 2005), yet simultaneous detection of δ-ALA and PBG is essential for the differential screening of the aforementioned conditions.

The classical method for the qualitative determination of urinary PBG constitutes the colorimetric analysis after reaction with Ehrlich’s reagent (p-dimethylaminobenzaldehyde, DMAB) in acid solution forming a red compound. In this process, equal amounts of urine and Ehrlich’s reagent are mixed and PBG is obtained in the aqueous phase (Watson and schwartz, 1941). This method was later revised and simplified by Hoesch (Lamon and Redeker, 1974). However, these methods lack adequate selectivity and sensitivity for quantitative measurements, as the interfering compounds are extracted from the urinary sample before initiating the reaction. As the most prevalent alternative, Mauzerall and Granick’s method allows the quantification of both δ-ALA and PBG with the help of multiple sample extraction steps. In this method, urinary samples are purified by using different cation and anion-exchange columns before derivatization with Ehrlich’s reagent and precise spectrophotometric determination at 553 nm (Mauzerall and Granick, 1956). Driven by these methods, a plethora of assays (Zhang et al., 2011; Benton et al., 2012; Carichon et al., 2014) have been introduced to achieve simple and speedy quantification of δ-ALA and PBG. Despite their huge contribution towards the detection, the existing screening methods still meet the challenge to separately determine δ-ALA and PBG in complex environments with interference from plentiful coexisting compounds reacting with Ehrlich’s reagent (Marver et al., 1966; Pierach et al., 1977). Therefore, complicated and lengthy purification processes to reduce background interference from coexisting components become unavoidable, which deprives most hospitals of rapid detection for acute porphyria in patients with neuropathic abdominal issues. This delay usually results in awfully misdirected treatment (Bissell, 2015). Clearly, a simple, rapid and reliable method for direct and simultaneous determination of δ-ALA and PBG is of important significance.

Chemical derivatization is widely used to enhance the detection sensitivity and retain δ-ALA and/or PBG on columns in chromatography-based approaches (Hijaz and Killiny, 2016). Modified Hantzsch reaction is reckoned as one of the most frequently employed derivatization agent for δ-ALA, as it entails the detection based on fluorescence (Tomokuni et al., 1992; Oishi et al., 1996; Donnelly et al., 2006; Kanto et al., 2013). Fluorescence spectrometry has been a powerful tool for the identification, monitoring, and quantification of various analytes in a broad range of environments, on account of high selectivity and sensitivity (Ou-Yang et al., 2018; Tsumura et al., 2018; Cheng et al., 2019; Zhai et al., 2020; Ding et al., 2022; Remolina et al., 2022; Wang et al., 2022). Despite the extensive use, the considerable background fluorescence and the spectral overlap contributed by the interfering components are liable to pose a formidable diagnostic challenge for conventional fluorescence spectrometry in case of complex multi-component analysis. The technological advancement of synchronous fluorescence spectrometry (SFS) in biological applications shows great promise in solving this problem by taking advantage of spectral simplification, band narrowing, and minimized scattering interference (Lecrenier et al., 2018; Elmas et al., 2019; Kumar, 2019; Ouyang et al., 2019; Zhao et al., 2020). SFS exploits simultaneous scanning for excitation and emission wavelengths. With the passage of time, SFS has been transformed into a number of branches, including conventional constant-wavelength SFS (defined with constant Δλ), constant-energy SFS, variable angle SFS, and matrix-isopotential SFS. Constant-energy SFS deals with maintaining a constant energy difference of the excitation and emission photons (Li et al., 2010). In variable-angle SFS, the excitation and emission wavelengths are varied simultaneously but at different rates (Samokhvalov, 2020).

Matrix-isopotential synchronous fluorescence spectrometry (MISFS), a branch of SFS, specializes in eliminating the unknown fluorescence matrix background and determining the individual compounds in complex matrices simultaneously (Pulgarín and Molina, 1994). This technique proceeds by establishing a cut in the combined fluorescence spectrum for maintaining a constant matrix background. Subsequently, the cut is accompanied by the matrix-isopotential trajectory, which joins together the points of equal fluorescence intensity. Most importantly, as the selected trajectory passes all through the emission and excitation wavelength maxima of the target components in the matrix, the sensitivity is increased markedly (Li et al., 2010). Derivative spectrofluorimetry serves as an analytical tool which can transform the normal spectrum of a spectral curve into a derivative spectrum. Finally, MISFS in combination with the first derivative technique emerges as an ideal platform for multi-component detections by minimizing the unknown and unspecific background fluorescence from urine, and by resolving the spectra of adjacently overlapping mixtures, without resorting to pre-analytical purification processes (Pulgarín et al., 2014).

Capitalizing on our previous knowledge regarding the porphyrins and the porphyria (Huang et al., 2010; Shindi et al., 2010; Liu et al., 2012; Ajmal et al., 2019), we combine derivative matrix-isopotential synchronous fluorescence spectrometry (DMISFS) and the Hantzsch reaction to eliminate the long-standing problems of interference and laborious pre-analytical purifications for the simple, speedy and simultaneous determination of δ-ALA and PBG in the urine. While it is common to use the Hantzsch reaction for the fluorescent detection of δ-ALA, to our knowledge, there exists no precedent for either the detection of PBG alone or simultaneously with δ-ALA involving Hantzsch reaction or DMISFS. Thus, our work is envisioned to serve as a novel diagnostic tool for patients with severe abdominal pain in both clinical and scientific applications. This work also displays great promise as potential alternative to the present day kits for quantitative detection of δ-ALA/PBG.

## Experimental

### Materials and Reagents

δ-ALA hydrochloride, acetylacetone reagent, and Formaldehyde (37%, V/V in water) were provided by Aladdin-Reagent (Shanghai, China). Porphobilinogen was purchased from Sigma-Aldrich (St. Louis, MO, United States). Methanol HPLC grade, glacial acetic acid, and hydrochloric acid were supplied by J&K (China). The aqueous solutions were prepared by purified water. The ultrapure water was obtained from a Millipore Milli-Q water purifying system (Millipore, Bedford, MA, United States), with a specific resistance of 18.2 MΩ cm.

Stock solutions for δ-ALA and PBG were prepared as 3 and 1 mM, respectively, in amber-colored volumetric flasks and were preserved in the refrigerator until use. Samples for both the analytes were prepared following a procedure reported previously (Ajmal et al., 2019). Calibration plots were obtained by spiking different solutions with concentrations as 0–5 µM for δ-ALA and 0–15 µM for PBG. Acetylacetone reagent was formulated by mixing acetylacetone, ethanol, and water in a ratio of 15:10:75, respectively. Formaldehyde solution was prepared by 3.7–fold dilution of commercially available formaldehyde solution and was stored in darkness (Oishi et al., 1996).

### Urine Samples

Urine specimens from apparently healthy individuals were collected, anonymized, stored in dark containers, and refrigerated at −20°C. The obtained samples were generally analyzed within an hour from their collection time. This research was carried out according to the regulations and guidelines of the National Ethics Committee for the Biomedical Research in China. All experiments complied with the institutional guidelines and relevant laws approved by the Medical Ethics Committee of Xiamen University. Informed consent was obtained from the volunteers participating in this study.

### Fluorescence Study

All the fluorimetric experiments were conducted on a laboratory-assembled MYF spectrofluorometer (Huang et al., 2010; Liu et al., 2016; Ajmal et al., 2019). The instrument was equipped with 150-W xenon lamp as the excitation source. For the two monochromators, the slits were fixed at 5 nm band passes. To control the monochromators and obtain the excitation and emission spectra, a software package framed in Turbo C 2.0 was used. This software permits the export of the obtained fluorescence data into the format of ASCII file. ASCII file is employed in the FTOTAL program, which is written on Visual BASIC (Pulgarín and Molina, 1993). This program favors the representation of three-dimensional (3D) spectra of the individual compounds from their corresponding excitation and emission spectra. These 3D spectra can also be displayed as a contour map or an isometric projection. For recording the MISF spectrum, FTOTAL achieves the intensity values for the excitation wavelengths by applying the specific function which combines points of identical intensity on the matrix comprehensive fluorescence spectrum (Wu et al., 1999). This combination of equal intensity points is called the isopotential scanning trajectory. The first derivative spectra were then acquired directly via electronic differential device equipped with the spectrofluorometer without a phase converter. A quartz cuvette with a path length of 1.0 × 1.0 cm was utilized for all the fluorescence measurements.

## Results and Discussion

### Conventional Fluorescence Spectra

**FIGURE 1:**
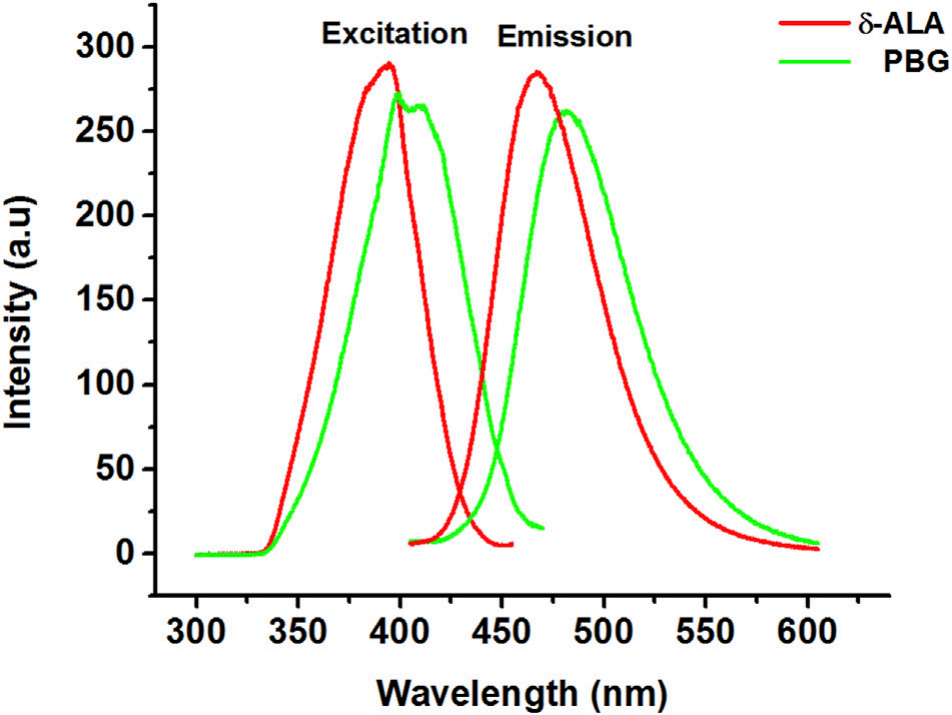
Excitation and emission spectra of δ-ALA (2.5 μmol L^−1^, red solid lines, λex = 394.4 nm; λem = 467.3 nm) and PBG (6.5 μmol L^−1^, green solid lines, λex = 399.7 nm; λem = 481.6 nm).

We commenced our study by obtaining conventional fluorescence spectra for δ-ALA and PBG. Figure 1 exhibits the location of the excitation maxima of δ-ALA and PBG at 394.4 and 399.7 nm, respectively. And the corresponding emission maxima of δ-ALA and PBG at 467.3 and 481.6 nm, respectively. However, in this case, fluorescence bands of δ-ALA and PBG were closely overlapped on account of their almost identical molecular structures, indicating the fact that the presence of one component would overwhelmingly influence the other and hence their simultaneous quantitative detection using the conventional fluorescence spectrometry might well be difficult without resorting to the tedious and lengthy pre-separation processes.

### Total Fluorescence Spectra

**FIGURE 2:**
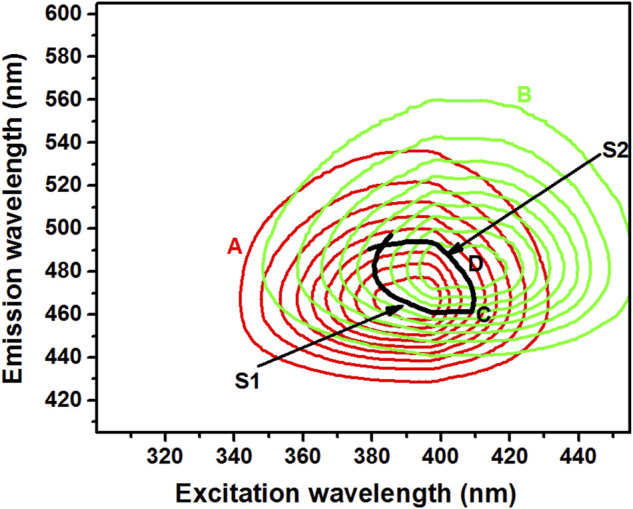
Contour maps of δ-ALA [**(A)**, 2.5 μmol L^−1^ *,* red solid line], PBG [**(B)**, 6.5 μmol L^−1^ *,* green solid line], point of contact **(C)** and the selected isopotential scanning route [**(D)**, black solid line].

Theoretical three-dimensional (3D) spectra for both δ-ALA and PBG were obtained for selecting suitable scanning path of MISF. 3D spectra not only provide wide scope and comprehensive structural information, but can also furnish spectral features invisible in the conventional fluorescence spectra. These spectra were extracted from pair of emission and excitation spectra of both δ-ALA and PBG by using a home-made program (FTOTAL) and projected as the isometric presentation, where the emission spectra were plotted at corresponding increments of the excitation wavelengths. Subsequently, 3D spectra were efficiently transformed into two-dimensional contour plots by connecting the points of identical fluorescence intensity of the emission and excitation wavelengths. As shown in Figure 2, the superposition of the contour plots of δ-ALA and PBG leads to the overlapping in the maximum intensity regions of the analytes. This serious overlap disabled the individual detections owing to the spectral interference from the background signal.

### Optimum Scanning Route Selection

The careful evaluation of the 2D contour plots of δ-ALA and PBG favored the selection of an optimum matrix-isopotential scanning trajectory by making a cut in the total fluorescence spectra of the two analytes. The selection of this trajectory was critically significant in establishing the suitable DMISFS method for the rapid and simultaneous detection of δ-ALA and PBG, since it could offer the DMISF spectra with the maximum fluorescence and minimum interference. In the selected trajectory, from left to right, the first section (S1) was a part of PBG contour lines, while the second section (S2) belonged to δ-ALA contour lines. Section1 (PBG) of the isopotential trajectory was chosen to pass through the highest possible wavelengths of δ-ALA: ex = 391 nm, em = 465.4 nm. Similarly, section 2 (δ-ALA), was selected to go through the highest possible wavelengths of PBG: ex = 403.6 nm, em = 484.3 nm. Later, these sections were combined at the crossing point, highlighted by the “C” sign to establish a complete scanning path as a matrix isopotential synchronous fluorescence (MISF) scanning route for the simultaneous detection of δ-ALA and PBG (Figure 2). The trajectory was highlighted by the sign “D”. This isopotential scanning route was used for both MISF and DMISF based detections. When this selected path was used for acquiring luminescence spectra of δ-ALA and PBG in the presence of urinary fluorescent matrix, the same spectrum was obtained for the targets as in complete isolation, thereby, maintaining high sensitivity.

**FIGURE 3:**
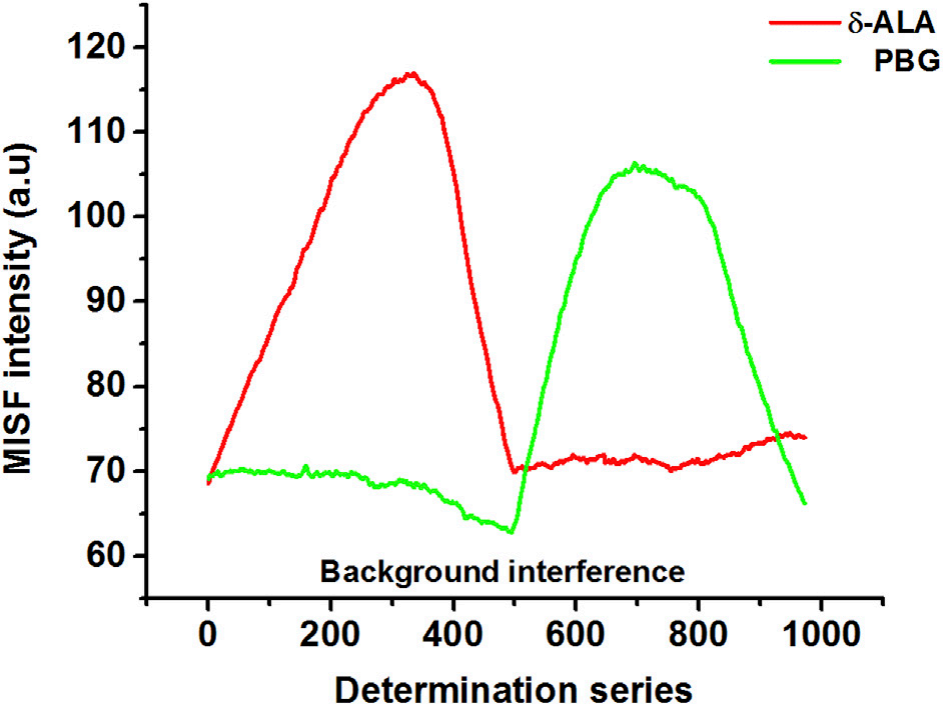
MISF spectra of δ-ALA (2.5 μmol L^−1^, red solid line) and PBG (6.5 μmol L^−1^, green solid line)*.*

Figure 3 shows MISF spectra for δ-ALA and PBG. For appropriate presentation and quantitative determinations, the MISF spectra were outlined with the determination series plotted as the *x*-axis, while the MISF intensity set as the *y*-axis. Moreover, the determination series demonstrate the coordinate orders of all the positions (ex, em) employed for the selection of the isopotential scanning path, beginning from the extreme left up to the extreme right.

**FIGURE 4:**
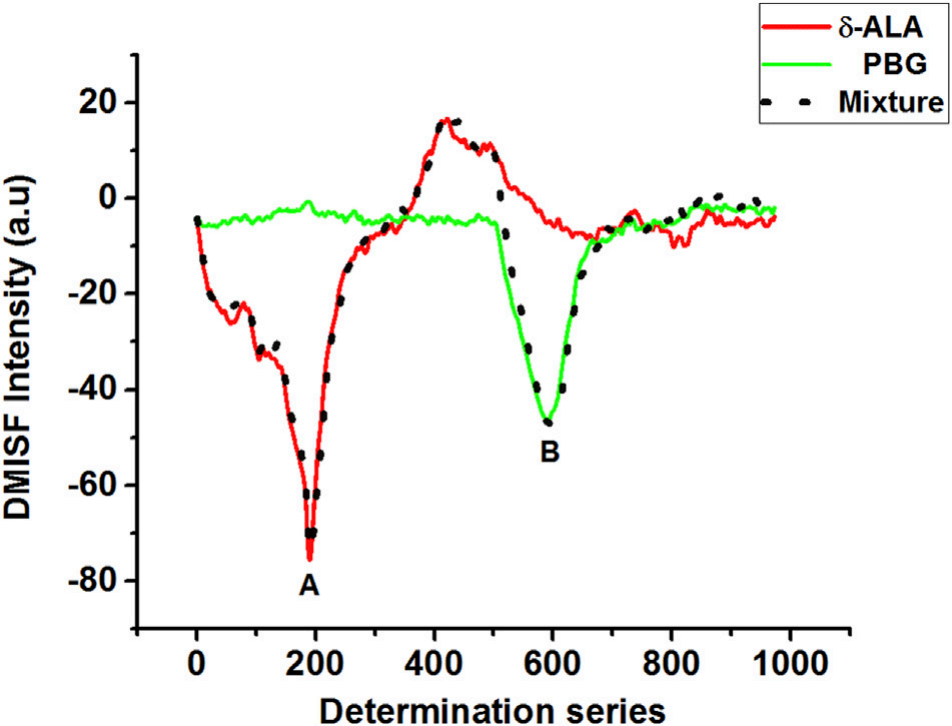
DMISF spectra of δ-ALA (2.5 μmol L^−1^, red solid line), PBG (6.5 μmol L^−1^, green solid line), and their mixture, black dotted line. **(A,B)** refer to the detection points of δ-ALA and PBG respectively.

Although the spectral bands of δ-ALA and PBG were satisfactorily resolved, their quantitative measurement using MISFS technique alone might still not be precise owing to the background interference of one component on the other. First-derivative technique has been an effective tool of background elimination and spectrum enhancement, which can improve resolution ability of fine spectral structures and identify subtle spectral changes. Considering the fact that the scanning path was selected isopotentially, this issue was resolved by using the first-derivative technique in which the baseline involvement of both analytes in the normal MISF spectrum offered no interference in the first-derivative MISF spectrum. Coupling MISF technique with the first-derivative, it was possible to resolve the interference caused by the spectral overlap and determine both δ-ALA and PBG separately with high sensitivity and selectivity (Figure 4). Figure 4 displays the elimination of PBG signal in the first section while retaining the net-derivative signal of δ-ALA, and vice versa for the second section. DMISF spectra of δ-ALA and PBG were scanned by the selected scanning path. The measurements corresponding to the peak maxima in the DMISF spectra of δ-ALA and PBG, highlighted as “A and B” on Figure 4, were applied for setting up their calibration plots.

**FIGURE 5:**
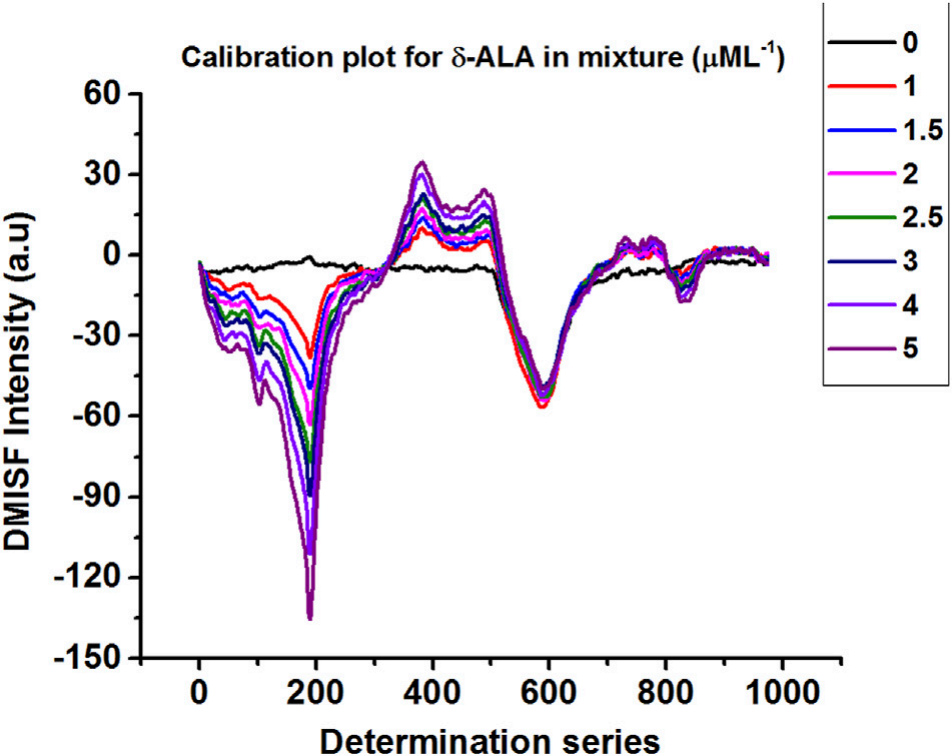
DMISF spectra of 8 μmol L^−1^ PBG with increasing amount of δ-ALA 0–5 μmol L^−1^.

**FIGURE 6:**
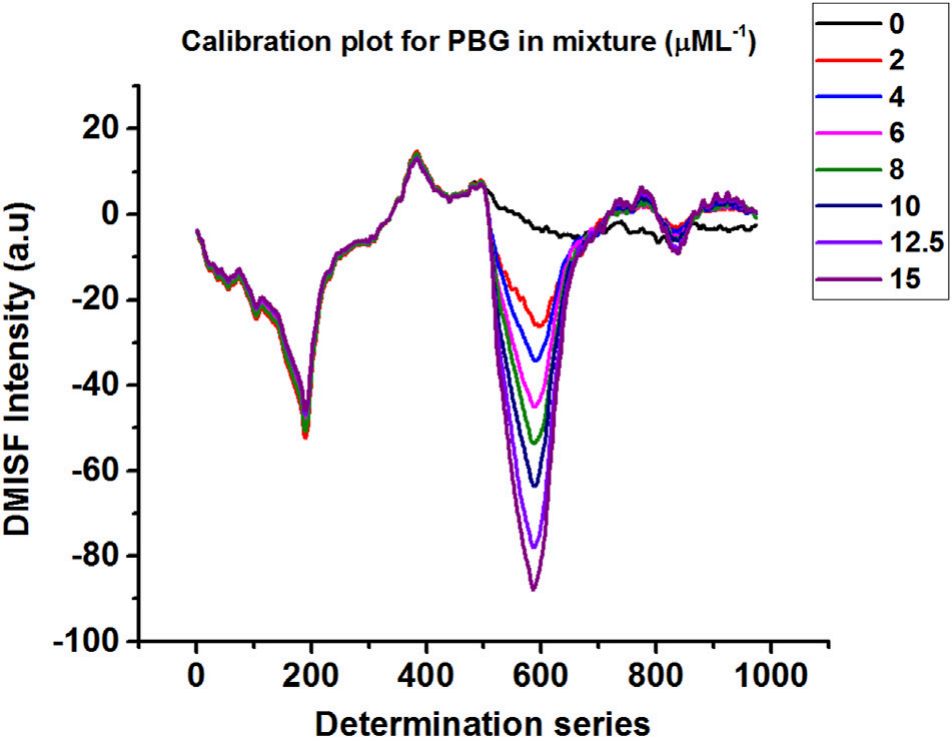
DMISF spectra of 1.5 μmol L^−1^ δ-ALA with increasing amount of PBG 0–15 μmol L^−1^.

Calibration plots for both δ-ALA and PBG were constructed by diluting appropriate aliquots of each analyte to verify their mutual independence at the selected detection points. The independence of δ-ALA detection point was displayed by obtaining DMISF spectra in mixtures having 0–5 μmol L^−1^ δ-ALA while the PBG concentration was fixed as 8 μmol L^−1^. Likewise, the independence of PBG detection point was exhibited by conducting the DMISF spectra in mixtures with 0–15 μmol L^−1^ PBG while δ-ALA concentration was fixed, as 1.5 μmol L^−1^ (Figures 5, 6). As portrayed by Figures 5, 6, the DMISF spectra for δ-ALA and PBG at their respective detection points were mutually independent and well resolved, thereby, could well be quantified simultaneously in just a single scan within 90s. Moreover, the calibration plots were prepared by employing the linear regression method. The relationship between the analyte concentration and the DMISF signal at their respective detection points was linear up to the concentrations of 5 and 15 μmol L^−1^ for δ-ALA and PBG, respectively. The achieved correlation coefficient, 0.998 for both the analytes separately and individually, indicated good linearity.

### Limits of Detection and Quantification

In compliance with IUPAC definition, the limits of detection were estimated as 0.04 and 0.31 μmol L^−1^ for δ-ALA and PBG respectively. The results were obtained with the equation C_LOD_ = 3Sb/m, where Sb symbolizes the standard deviation of the blank measurements (*n* = 16) and m refers to the slope of the calibration curve for the corresponding analyte. For the limits of quantification, the equation 10Sb/m, was used. The limits of quantification was found as 0.15 μmol L^−1^ for δ-ALA and 1.04 μmol L^−1^ for PBG. The detection limits achieved by the proposed method are satisfactory for the routine analysis of δ-ALA and PBG in urine samples.

### Application of the Method to Spiked Urine Samples

In order to investigate the potentiality of the proposed method towards the quantitative determination of δ-ALA and PBG, the DMISF spectra for a number of urine samples spiked with the target analytes with contrasting ratios were obtained at the isopotential scanning path. Previously constructed calibration plots were used for obtaining the concentrations of both components. The findings of these assays indicate the applicability of the present method for the precise quantification of δ-ALA and PBG in their mixtures (Table 1, Supplementary Figures S1A–E, ESI).

**TABLE 1:** Urine samples spiked with δ-ALA and PBG and obtained concentrations.

Sample no.	Spiked ALA (µmol L^−1^)	Spike PBG (µmol L^−1^)	Found ALA (µmol L^−1^)	Found PBG (µmol L^−1^)
1	1	6	1.0	5.0
2	3	11	2.8	9.7
3	1	10	0.9	8.8
4	3	4	2.8	2.8
5	4	7	3.8	5.5

Here, the found concentrations refer to the net concentrations of δ-ALA and PBG obtained after subtracting the signal of unspiked urine samples**.**

### Precision of the Method

The within-run precision for the present method was demonstrated by repeatedly analyzing δ-ALA and PBG in a randomly obtained urine sample, from an apparently healthy subject. To be precise, the random urinary sample was analyzed for intra-day CVs (ten times a day) and inter-day CVs (six times during 6 days). The used sample was stored at −20°C. The between-run precision for intra-day CVs turned out be 3.0 and 3.1% for δ-ALA and PBG, respectively (Supplementary Figure S2A, ESI). However for inter-day, the CVs were 4.3 and 4.0% for δ-ALA and PBG, respectively (Supplementary Figure S2B, ESI).

## Conclusion

Based on the combination of Hantzsch reaction, MISFS and the first-derivative technique, a direct and straightforward strategy was proposed aiming at the simultaneous quantification of δ-ALA and PBG in urinary samples for early diagnosis and treatment of acute porphyria. Compared with the previously reported methods, DMISFS effectively removed the spectral overlap and minimized the influence of unknown background fluorescence, making a seminal contribution as a highly sensitive technique which demanded no pre-analytical purification processes, no complicated operations and could rapidly quantify both the analytes in a single scanning within 90 s. Moreover, the present method offered improved recovery, selectivity, and limits of detections. The present method was also examined for the detection of δ-ALA and PBG in urine samples without any significant interference. This work stands out as a successful effort to not only furnish a reliable alternative for the laboratory-based diagnosis of δ-ALA and PBG but also for the commercially available screening kits with a number of added advantages. The present method is also believed to strengthen the international strives against the acute porphyria by providing prompt analysis of δ-ALA and PBG.

## Data Availability

The original contributions presented in the study are included in the article/Supplementary Material, further inquiries can be directed to the corresponding author.
