# Supplementary material for: Derivative Matrix-Isopotential Synchronous Spectrofluorimetry and Hantzsch Reaction: A Direct Route to Simultaneous Determination of Urinary δ-Aminolevulinic Acid and Porphobilinogen
Source: Front Chem. 2022 May 31;10:920468. doi: 10.3389/fchem.2022.920468 (PMC9194443; doi:10.3389/fchem.2022.920468)
Supplement: Supplementary file 1 [file DataSheet1.DOCX]

**1. Urine Samples**

The applicability of the method for the quantitative determination of ALA and PBG was tested by scanning the DMISF spectra of several urine samples spiked with ALA and PBG with different ratios. The obtained concentrations were compared with the established calibration curve. The results of assaying ALA and PBG mixtures indicate good accuracy for their determination as mixtures (Fig. S1 a-e).















**Fig. S1** DMISF Spectra for unspiked and spiked urine samples (1-5)

**2. Inter and Intra-day CVs**

The within-run precision of the method (Fig. S2) was assessed by repeatedly assaying a random urinary sample from apparently healthy volunteer for intra-day CVs (ten times a day) and inter-day CVs (six times in six days). The sample was stored at −20 ^0^C and the between-run CVs were acquired as 3.0% and 3.1% of intra-day for ALA and PBG. However for inter-day, the CVs were 4.3% and 4.0% for ALA and PBG, respectively.







**Fig. S2** DMISF Spectra for Intra-day (a) and Inter-day (b) CVs
